# ADSCs labeled with SPIONs tracked in corpus cavernosum of rat and miniature pig by MR imaging and histological examination

**DOI:** 10.1038/s41598-023-51076-2

**Published:** 2024-01-22

**Authors:** Qingqiang Gao, Jianhuai Chen, Wenren Zuo, Bin Wang, Tao Song, Chunlu Xu, Wen Yu, Yutian Dai, Songzhan Gao, Leilei Zhu, Jie Yang

**Affiliations:** 1 Department of Andrology, Nanjing Drum Tower Hospital, The Affiliated Hospital of Nanjing University Medical School, Nanjing, Jiangsu China; 2 Department of Andrology, Jiangsu Province Hospital of Chinese Medicine, Affiliated Hospital of Nanjing University of Chinese Medicine, Nanjing, China; 3 Department of Urology, Jiangsu Province Hospital of Chinese Medicine, Affiliated Hospital of Nanjing University of Chinese Medicine, Nanjing, China; 4 Department of Andrology, The Third Affiliated Hospital of Zhengzhou University, Zhengzhou, China; 5 Department of Urology, The Affiliated Wuxi People’s Hospital of Nanjing Medical University, Wuxi People’s Hospital, Wuxi Medical Center, Nanjing Medical University, Wuxi, Jiangsu China; 6 Department of Surgery, Aheqi County People’s Hospital, Xinjiang, China; 7 Department of Urology, Jiangsu Provincial People’s Hospital, First Affiliated Hospital of Nanjing Medical University, Nanjing, China; 8 Department of Urology, People’s Hospital of Xinjiang Kizilsu Kirgiz Autonomous Prefecture, Xinjiang, Uygur Autonomous Region China

**Keywords:** Stem cells, Urology

## Abstract

Adipose tissue-derived stem cells (ADSCs) have been shown to improve erectile function in animal models of erectile dysfunction. However, few studies have been carried out using a reliable in vivo imaging method to trace transplanted cells in real time, which is necessary for systematic investigation of cell therapy. The study aims to explore the feasibility of non-invasively monitoring intracavernous injection of ADSCs in rat and miniature pig corpus cavernosum using in vivo magnetic resonance (MR) imaging. Thirty-six male Sprague Dawley rats (10 weeks old) and six healthy, sexually mature male miniature pigs (20 kg weight) were obtained. ADSCs were isolated from paratesticular fat of donor rats and cultured. Then ADSCs were labeled with superparamagnetic iron oxide nanoparticles (SPIONs), a type of MR imaging contrast agent, before transplantation into rats and pigs. After intracavernous injection, all rats and pigs underwent and were analyzed by MR imaging at the day of ADSC transplantation and follow-up at 1, 2 and 4 weeks after transplantation. In addition, penile histological examination was performed on all rats and pigs before (n = 6) and at 1 day (n = 6), 1 week (n = 6), 2 weeks (n = 6) or 4 weeks (n = 12) after ADSC transplantation. SPION-labeled ADSCs demonstrated a strong decreased signal intensity compared with distilled water, unlabeled ADSCs or agarose gel. SPION-labeled ADSCs showed a hypointense signal at all concentrations, and the greatest hypointense signal was observed at the concentration of 1 × 10^6^. MR images of the corpus cavernosum showed a hypointense signal located at the injection site. T2*-weighted signal intensity increased over the course of 1 week after ADSCs transplantation, and demonstrated a similar MR signal with that before ADSCs transplantation. After SPION-labeled ADSC injection, T2*-weighted MR imaging clearly demonstrated a marked hypointense signal in pig corpus cavernosum. The T2*-weighted signal faded over time, similar to the MR imaging results in rats. Obvious acute inflammatory exudation was induced by intracavernous injection, and the T2*-weighted signal intensity of these exudation was higher than that of the injection site. The presence of iron was detected by Prussian blue staining, which demonstrated ADSC retention in rat corpus cavernosum. Lack of cellular infiltrations were demonstrated by H&E staining before and 4 weeks after transplantation, which indicated no negative immune response by rats. Prussian blue staining was positive for iron oxide nanoparticles at 2 weeks after transplantation. SPION-labeled ADSCs showed a clear hypointense signal on T2-weight MRI in vitro and in vivo. The MR signal intensity in the corpus cavernosum of the rats and miniature pigs faded and disappeared over time after ADSC transplantation. These findings suggested that MR imaging could trace transplanted ADSCs in the short term in the corpus cavernosum of animals.

## Introduction

Stem cell therapy is a promising new frontier for the treatment of refractory erectile dysfunction (ED) and many studies have demonstrated its therapeutic effects. However, few studies have been carried out using a reliable in vivo imaging method to trace transplanted cells in real time, which is necessary for systematic investigation of cell therapy. Superparamagnetic iron oxide nanoparticle (SPION) as a type of conventional MR imaging contrast agent can produce a strong signal on magnetic resonance (MR) images.

Adipose tissue-derived stem cells (ADSCs) exhibited a stronger proliferation ability and multidifferentiation potential. However, the differentiation of SPION-labeled ADSCs after intracavernous injection is still a major issue in stem cell transplantation. In our previous study^1^, we demonstrated that SPIONs could be effectively incorporated into ADSCs without influencing ADSC proliferation or viability in vitro. Moreover, SPIONs have been studied extensively for stem cell labeling in recent years^2,3^, as MR imaging is a noninvasive and serial consecutive imaging method that can provide high-resolution and -sensitivity information for transplanted cells. Moreover, we and other researchers have employed SPIONs to label stem cells and guide them to target areas by magnetic field application^1,4–6^. The biological activity and MR imaging of ADSCs by labeling them with SPIOs were performed in previous study^7^. The results showed that ADSCs could be labeled and traced easily in vitro. Given their abundance and higher proliferative capacity, ADSCs were considered to be better suited to stem cell therapy. In another study, ADSCs were efficiently labeled with SPIONs, without affecting their viability and proliferation^8^. The labeled cells implanted into the mice exhibited a significant increase in regeneration and could be detected by MRI. Moreover, fluorescent microscopic examination, histological analysis and immunohistochemistry confirmed the axon regeneration and MR imaging results. All these findings suggested that ADSCs showed great promise in the regenerative repair of injured tissue and MR imaging has provided attractive advantages in tracking SPIOs-labeled cells and evaluating their fate after cell transplantation. However, the method of MR imaging has not been used to trace cells in the corpus cavernosum in animal models.

The goal of this study was to tracking ADSCs labeled with SPIONs in corpus cavernosum of rat and miniature pig by MR imaging. Furthermore, retention of ADSCs in rat corpus cavernosum was confirmed by histological examination.

## Materials and methods

### Animals

Thirty-six male Sprague Dawley rats (10 weeks old) and six healthy, sexually mature male miniature pigs (20 kg weight) were obtained from the Animal Breeding Center at our hospital. The experiments were approved by the Institutional Animal Care and Use Subcommittee of Nanjing Drum Tower Hospital, The Affiliated Hospital of Nanjing University Medical School. Moreover, this study was reported in accordance with ARRIVE guidelines.

### Preparation of ADSCs

ADSCs were isolated from paratesticular fat of donor rats and cultured as described previously^1^. Surface markers were identified by flow cytometric analysis of passage 3 ADSCs. Briefly, the adipose tissue was rinsed with PBS containing 1% penicillin and streptomycin on ice, minced into small pieces, and then incubated in a solution containing 0.075% collagenase type IA (Sigma-Aldrich, St. Louis, MO) for 1 h at 37 °C with vigorous shake. The top lipid layer was removed and the remaining liquid portion was centrifuged at 220 g for 10 min at room temperature. The pellet was treated with 160 mM NH4Cl for 10 min to lyse red blood cells. The remaining cells were suspended in low-glucose DMEM (Hyclone, Thermo Science, San Jose, CA, USA) supplemented with 10% FBS (Invitrogen, Carlsbad, CA, USA) in a standard 5.0% CO_2_ incubator at 37 °C. Passage 3 ADSCs from multiple donors were used in these experiments.

### SPIONs labelling

As described previously^1^, passage 3 ADSCs were seeded in culture plates and cultured with the complete medium containing SPIONs (Sigma-Aldrich, St. Louis, MO, USA) at a final concentration of 50 µg Fe/mL for 24 h. After incubation, the medium was removed, and the cells were washed three times with PBS for use in further experiments.

### In vitro MR imaging

Transverse MR images of SPION-labeled ADSCs in vitro using a RARE-T2-weighted sequence were obtained with a horizontal bore 7.0-T scanner (Bruker PharmaScan, Ettlingen, Germany). Imaging parameters for in vitro scans: echo time (TE) = 33.0 ms, repetition time (TR) = 2500.0 ms, the field of view (FOV) = 4.0 cm × 4.0 cm, matrix = 256 × 256, in plane resolution = 156 μm × 156 μm, each imaging time was 1 min and 20 s. Increasing concentrations of labeled cells (1 × 10^4^ to 1 × 10^6^) in 2% agar were filled into tubes and used for T2 measurements. These measurements were compared with those for distilled water, agarose gel and unlabeled ADSCs.

### Intracavernous transplantation of SPION-labeled ADSCs

For in vivo experiments, intracavernous transplantation of SPION-labeled ADSCs was performed as described previously^1^. All rats were anesthetized with isoflurane, and a 1.5-cm oblique incision was made to expose the penis. The corpus cavernosum was then gently cannulated using a 28-gauge needle, and each rat received an injection of 1 × 10^6^ SPION-labeled ADSCs in 0.5 ml PBS into the left corpus cavernosum.

Preoperative fasting was done in six pigs for 12 h before surgery to prevent inhalational pneumonia during surgery. The surgical procedures were performed under general anesthesia using a combination of 6 mg/kg ketamine chloride and 0.6 mg/kg xylazine (intramuscular injection) before the experimental procedures. After adequate skin preparation and sterilization, a 5.0-cm lower abdominal midline incision was made to expose the penis. The corpus cavernosum was then gently cannulated using a 5 ml-injector, and each rat received an injection of 1 × 10^6^ SPION-labeled ADSCs in 1.0 ml PBS into the right corpus cavernosum.

Following the injection, the incision was closed in one layer with an absorbable suture.

### In vivo MR imaging

Thirty-six rats and six pigs underwent MR imaging using a 7.0-T scanner (Bruker PharmaScan) at the day of ADSC transplantation and follow-up serial T2-weighted gradient-echo MR imaging at 1, 2 and 4 weeks after transplantation.

A spin echo sequence (TR500 ms, TE15 ms) was used for T1-weighted imaging and a FLASH sequence (TR2000 ms, TE50 ms) was used for T2*-weighted imaging. Imaging parameters for in vivo scans of rats: FOV = 6.0 cm × 6.0 cm, matrix = 256 × 256, in plane resolution = 234 μm × 234 μm, the slice thickness was 0.6 mm, each imaging time was 1 min and 44 s. Imaging parameters for in vivo scans of pigs: FOV = 25.0 cm × 18.0 cm, matrix = 256 × 256, in plane resolution = 977 μm × 703 μm, the slice thickness was 3 mm, each imaging time was 4 min and 48 s. The regions of interest (ROIs) were manually drawn on the penis area in the T1-weighted images.

### Histological examination

Rats were sacrificed before (n = 6) and at 1 day (n = 6), 1 week (n = 6), 2 weeks (n = 6) or 4 weeks (n = 12) after ADSC transplantation. The penises and sections were prepared as described previously by Song^9^. Briefly, the specimens were stained with standard hematoxylin and eosin (H&E) to observe the morphology of the penises. Prussian blue staining was performed to detect the presence of iron in histological sections.

### Ethics approval and consent to participate

The experiments were approved by the Institutional Animal Care and Use Subcommittee of Nanjing Drum Tower Hospital, The Affiliated Hospital of Nanjing University Medical School. In addition, experimental research on vertebrates or any regulated invertebrates were complied with institutional, national, or international guidelines, such as the Basel Declaration (https://animalresearchtomorrow.org/en).

## Results

### SPIONs labeling of ADSC leads to significant MR signal effects in vitro

**Figure 1:**
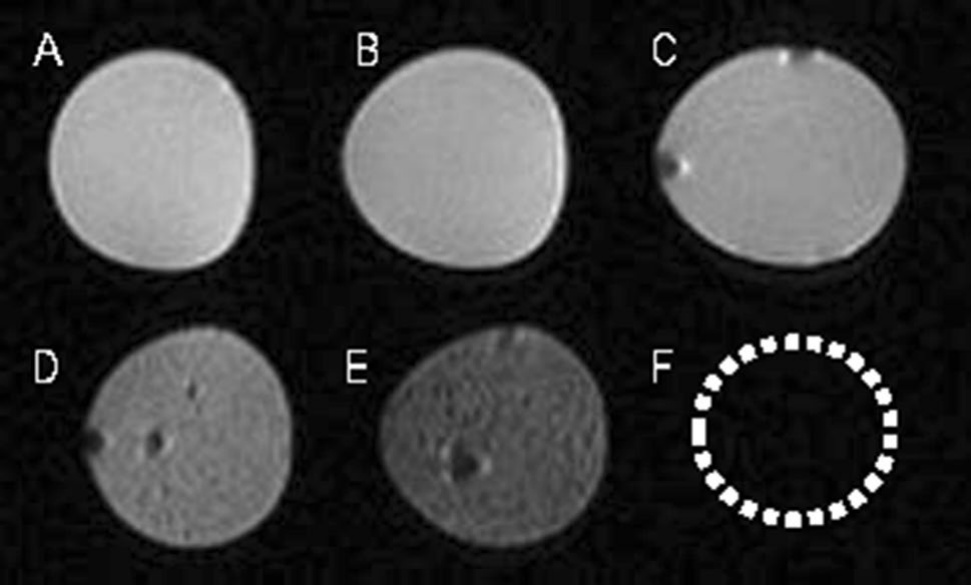
In vitro MR imaging of ADSCs. SPION-labeled ADSCs (**D–F**) produced a hypointense signal on T2*-weighted MR images compared with distilled water (**A**), agarose gel (**B**) and unlabeled ADSCs (**C**). SPION-labeled ADSCs at a concentration of 1 × 10^6^ (**F**) showed the greatest hypointense signal than 1 × 10^4^ (**D**) and 1 × 10^5^ (**E**). The in-plane resolution was 156 μm × 156 μm. The black spots were actually air bubbles that were not completely removed during the process of embedding cells in 2% agarose.

In vitro MR images of SPION-labeled ADSCs and controls were shown in Fig. 1. Overall, SPION-labeled ADSCs demonstrated a strong decreased signal intensity compared with distilled water, unlabeled ADSCs or agarose gel. In vitro MR images of SPION-labeled ADSCs at different concentrations were performed to assess the sensitivity of the MR imaging. SPION-labeled ADSCs showed a hypointense signal at all concentrations, and the greatest hypointense signal was observed at the concentration of 1 × 10^6^.

### SPION-labeled ADSCs can be detected in vivo with MR imaging

**Figure 2:**
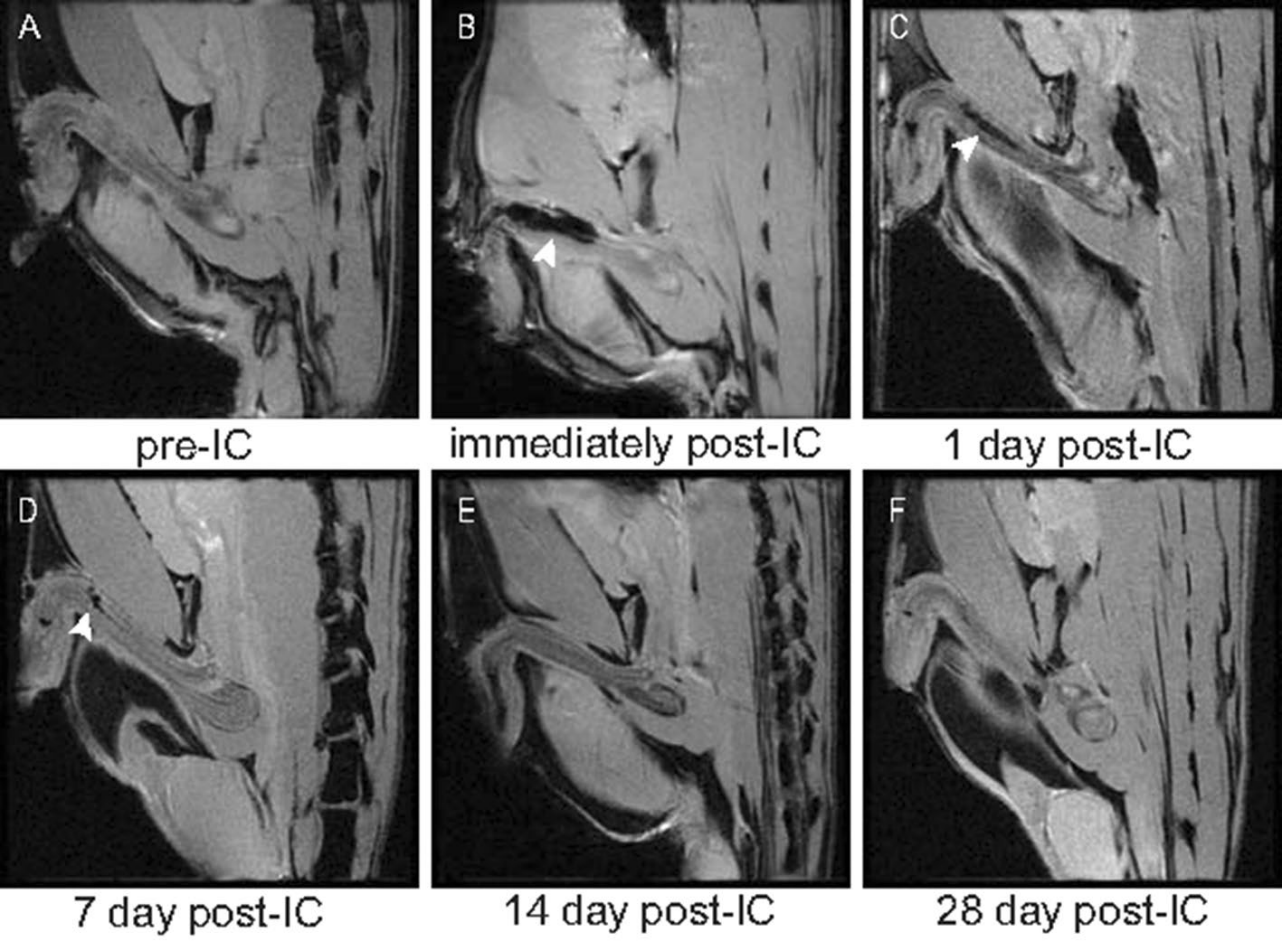
In vivo MR imaging of ADSCs in rat corpus cavernosum. The MR signal intensity in the corpus cavernosum of rats faded over time. (**A**) Before transplantation. (**B**) Immediately after transplantation. (**C**) At day 1 after transplantation. (**D**) At 1 week after transplantation. (**E**) At 2 weeks after transplantation. (**F**) At 4 weeks after transplantation. Arrow shows the decrease in MR signal intensity. The in-plane resolution was 234 μm × 234 μm.

MR images were taken immediately after SPION-labeled ADSCs transplantation, and MR images of the corpus cavernosum showed a hypointense signal located at the injection site (Fig. 2A, B). However, on follow-up studies, the T2*-weighted signal intensity increased over the course of 1 week after ADSCs transplantation, and demonstrated a similar MR signal with that before ADSCs transplantation (Fig. 2C–F).

**Figure 3:**
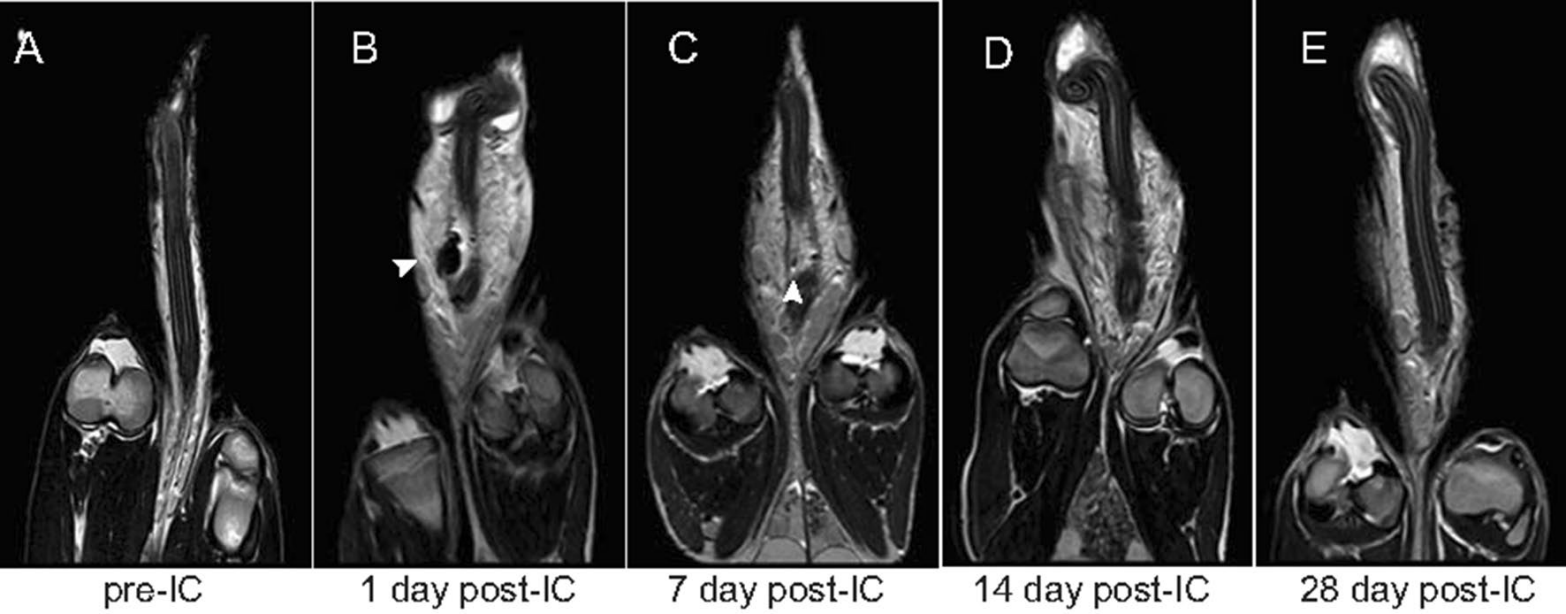
In vivo MR imaging of ADSCs in miniature pig corpus cavernosum. The MR signal intensity in the corpus cavernosum of pigs faded over time. (**A**) Before transplantation. (**B**) At day 1 after transplantation. (**C**) At 1 week after transplantation. (**D**) At 2 weeks after transplantation. (**E**) At 4 weeks after transplantation. Arrow shows the decrease in MR signal intensity. The in-plane resolution was 977 μm × 703 μm.

After SPION-labeled ADSC injection, T2*-weighted MR imaging clearly demonstrated a marked hypointense signal in pig corpus cavernosum (Fig. 3). The T2*-weighted signal faded over time, similar to the MR imaging results in rats. Obvious acute inflammatory exudation was induced by intracavernous injection, and the T2*-weighted signal intensity of these exudation was higher than that of the injection site. However, no serious infection or side effect happened, and exudation had been absorded completely at 4 weeks after intracavernous injection.

### Retention of ADSCs in rat corpus cavernosum can be further confirmed by Prussian blue staining

**Figure 4:**
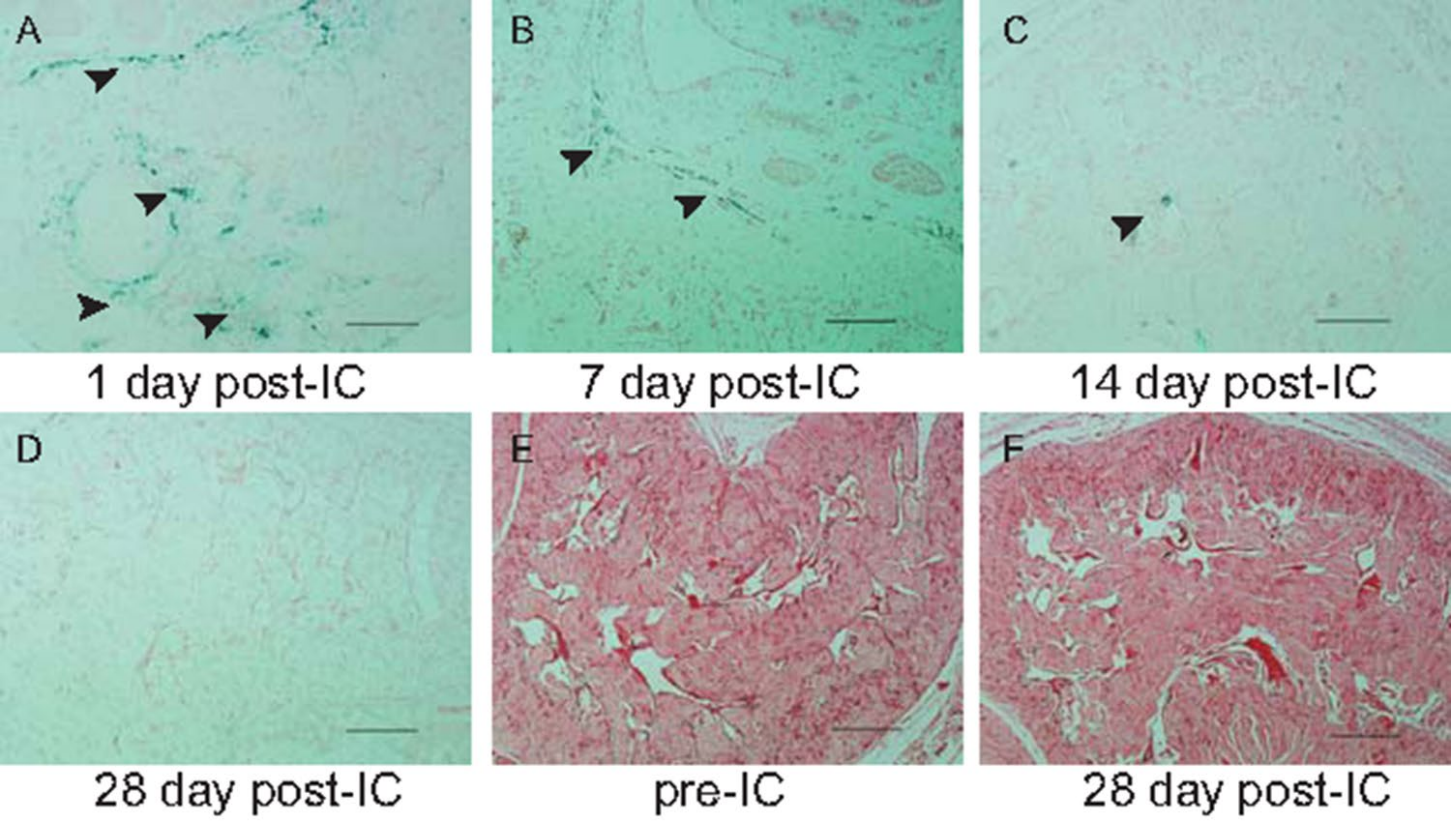
Histological examination of rat corpus cavernosum. At the end of each of the in vivo MR imaging experiments, SPION particles (blue dots) are clearly visible with Prussian blue staining (arrows). (**A**) At day 1 after transplantation. (**B**) At 1 week after transplantation. (**C**) At 2 weeks after transplantation. (**D**) At 4 weeks after transplantation. H&E staining was carried out before (**E**) and 4 weeks after transplantation (**F**). Lack of cellular infiltrations indicates no negative immune response by rats. Scale bars = 20 µm.

Prussian blue staining was performed to detect the presence of iron, which demonstrated ADSC retention in rat corpus cavernosum (Fig. 4A–D). H&E staining was carried out before and 4 weeks after transplantation (Fig. 4E, F). Lack of cellular infiltrations indicated no negative immune response by rats. Prussian blue staining was positive for iron oxide nanoparticles at 2 weeks after transplantation, which was contrary to the MR imaging data; this disparity was presumably a result of SPION dilution.

## Discussion

ADSCs are adult stem cells with great potential to differentiate into multilineage cells in vivo as well as in vitro. Our previous studies have shown the functional benefit of ADSC injection in erectile dysfunction models^1,10^. intracavernous injection is a locally applied intervention that is commonly thought to target the corpus cavernosum directly^11^. The penis is in fact a vascular organ, and intracavernous injection is similar to intravenous (IV) injection. It has been demonstrated that IV injection of ADSCs can restore erectile function in a rat model of radiation therapy-induced ED^10^. Lue et al.^12,13^ demonstrated that the majority of intracavernous injected stem cells exited the penis within 1 day, and preferentially traveled to the bone marrow or to the major pelvic ganglion in rats with cavernous nerve injury. It has been increasingly observed that the transplanted SCs did not necessarily engraft and differentiate at the site of injury but might exert their therapeutic effects through secreted trophic signals^14^.

However, the post-transplant complications commonly due to improper cell homing and engraftment. Therefore, tracing transplanted cells in vivo to understand the fate of these cells is necessary for systematic investigation of cell therapy. Unfortunately, none of most researchers’ attempts to find the transplanted cells in penile tissues has panned out, even though the animals clearly demonstrated functional and structural improvements. To monitor the distribution and survival of transplanted stem cells in penile tissues, most previous studies have used cells labeled with LacZ, DAPI, GFP, DiI, BrdU, or EdU^11^. Moreover, rather than allowing non-invasive in vivo imaging to trace transplanted cells in real time, animals in studies using these labeling methods had to be killed for histological examination.

In recent years, many studies have reported the feasibility of non-invasively monitoring the transplanted cells by MR imaging in vivo^15–17^. Such cell tracing method makes the transplanted cells directly visible for us, which is important for the development of cell therapy. In our current study, we found that ADSCs labeled with SPIONs could be detected in the corpus cavernosum with MR imaging; MR images of SPION-labeled ADSCs showed a strong hypointense signal compared with control groups, which included distilled water, unlabeled ADSCs and agarose gel. We also carried out in vitro MR images of SPION-labeled ADSCs at different concentrations to assess the sensitivity of the MR imaging. A clear hypointense signal at all concentrations was detectable, but the cells forming a cluster of 1 × 10^6^ or more showed the greatest hypointense signal on T2-weighted MR imaging, which indicated the optimal dose for in vivo studies. We therefore decided to use 1 × 10^6^ SPION-labeled ADSCs for in vivo studies monitoring their distribution in penile tissues. In this study, we confirmed MR imaging in vivo made us non-invasively monitor the labeled ADSCs in normal rat and pig corpus cavernosum.

It has been demonstrated that ADSCs lack major histocompatibility complex-II expression, and can control graft-versus-host disease^18^. Both preclinical and clinical studies have shown that allogeneic transplantation of ADSCs offers no obvious advantage over xenotransplantation^19^. In this study, we observed no evidence of immunological rejection, and confirmed that even xenogeneic ADSC transplantation was safe in vivo. The theoretical basis of our study was that SPION-labeled ADSCs showed a clear hypointense signal on T2*-weighted MR imaging, and the T2*-weighted sequence was more sensitive for the detection of iron than T2 weighted sequence. On follow-up studies, the hypointense signal intensity faded over time, and SPION-labeled ADSCs could be tracked over the course of 1 week after transplantation. Further, retention of ADSCs in rat corpus cavernosum was assessed by Prussian blue staining. Histopathology confirmed engraftment of labeled ADSCs, with slow dilution of the iron label over time. Mention that most studies based on an experimental rat model, we injected labeled cells into pig corpus cavernosum to simulate the fate of injected cells in clinical trials. The T2*-weighted signal intensity in pigs increased over time, similar to the MR imaging results in rats.

The data from this study showed that MR imaging can be used as a new approach to track ADSCs at least in the short term in rat and miniature pig corpus cavernosum. Despite all this, we still believe MR imaging is a promising approach to trace stem cells even in long term in the context of erectile dysfunction^20^. In condition of cavernous nerves injury models, several preclinical trials attempted to improve stem cells implantation by combining them with scaffolds (PLGA membrane, hydrogel or Matrixen)^21^ or cellular self-assembling into micro-tissues^22^. We speculate that MR imaging will fully demonstrate its advantages in tracking SCs under these condition^23–25^. However, Song et al.^9^ observed that the majority of injected cells remained at or near the injection site even 12 weeks after injection. The use of MR imaging to monitor injected cells in the corpus cavernosum is our preliminary study, which should be investigated further. MR imaging had several advantages for elucidating the fate of transplanted cells over other imaging modalities, such as the ability to image transplanted cells longitudinally at high spatial resolution without exposure to ionizing radiation, and the possibility to co-register anatomical structures with molecular processes and functional changes^26^. However, since MR imaging was still in its infancy, it currently faced a number of challenges. one major disadvantage was that the label itself was detected rather than the cells of interest^26^.

It was interesting to note that at day 1 after ADSC injection the corpus cavernosum showed a marked, hypointense T2*-weighted signal. This possibly resulted from the retention of the majority of labeled cells in the corpus cavernosum, as reported by Lue et al.^8,9^. Then the MR signal intensity faded over time, associated with the negative Prussian blue staining. Considered that the stability of SPION-labeled-ADSC should be checked until 28 days, it often needed to exclude the possibility that ADSCs were present due to natural decomposition but were not observable because they had been unlabeled. For ADSCs cultured in vitro, SPIONs would be excreted along with cell differentiation or via extracellular vesicles (EVs). Therefore, we did not investigate the stability of SPION-labeled ADSCs, and we boldly speculated that SPIONs would be mostly depleted in ADSCs cultured for 28 days. Of note, surface modification techniques for SPIONs had been proven to enhance stability of SPION-labeled-stem cells and improve the therapeutic effects of stem cells^27,28^. After injecting stem cells into the corpus cavernosum of the penis, less than 1% of the stem cells were able to remain in the corpus cavernosum, and this percentage continued to decrease over time. By the fourth week, the number of detectable SCs was extremely limited^29^. The majority of SCs belonged to the bone marrow^30^. Increasing evidence suggested that SCs did not necessarily need to differentiate into specific cell lineages in the injured area, but rather promote angiogenesis, inhibit apoptosis, or cell death, and regulate the immune system through paracrine or autocrine secretion of a series of cell-nourishing factors^14^.

This was our preliminary exploratory research, aimed at providing researchers with an option: MR imaging could be used in stem cell therapy to monitor the engraftment of stem cells more intuitively in local tissues, especially when using materials such as collagen or hydrogels to promote local engraftment of stem cells^31^. In vivo, SPIONs (a type of conventional MR imaging contrast agent) was typically taken up by phagocytic cells (macrophages and Kupffer cells), then cleared by the liver and spleen, and ultimately excreted in bile. Furthermore, whether SPIONs were involved in the iron metabolism pathways of cells and whether they affect the biological function of ADSCs would be further studied in our subsequent research. However, in our preliminary research, we had already explored the effective concentration of SPIONs labeled on ADSCs, and found that it had little effect on the proliferative activity of ADSCs. Moreover, SPION-labeled ADSCs could migrate and engraft under the influence of an external magnetic field in vitro^32^.

Due to technical issues such as injections into the subcutaneous tissue or urethra, or leakage of cell suspension, some of the MRI results in rats might not accurately reflect the engraftment status of ADSCs after corpus cavernosum injection. Therefore, we showed the representative photos. On the other hand, we were unable to perform quantitative analysis of the signal intensity for the specified region in MRI imaging, nor could we conduct quantitative analysis of the area for the designated region. Our experimental results visually demonstrated the engraftment of ADSCs after injection into the corpus cavernosum of rats, and we guaranteed the reproducibility of this experiment. In addition, in our previous research, we had confirmed that magnetic targeting SPIONs-labeled ADSCs could promote the engraftment of ADSCs in the corpus cavernosum and improve erectile function in diabetic rats. This study was a supplement to our previous research, aiming to explore the feasibility of using MR imaging for real-time monitoring SPION-labeled- ADSCs in the corpus cavernosum. However, considering the time and cost constraints associated with the 7.0 T MRI examination, we were unable to repeat the experiments using the ED rat model.

## Conclusions

In conclusion, this was the first study to explore the feasibility of non-invasively monitoring intracavernous injection of ADSCs in rat and miniature pig corpus cavernosum using in vivo MR imaging. The results demonstrated that SPION-labeled ADSCs showed a clear hypointense signal on T2 weighted MR imaging both in vitro and in vivo. The MR signal intensity in the corpus cavernosum of the rats and miniature pigs faded over a 1-week period and disappeared completely at day 7 after ADSC transplantation. These findings suggested that MR imaging could trace transplanted ADSCs in the short term in the corpus cavernosum of animals.

## Data Availability

The datasets used or analysed during the current study are available from the corresponding author on reasonable request.
